# Predictors of response to fixed-dose vasopressin in adult patients with septic shock

**DOI:** 10.1186/s13613-018-0379-5

**Published:** 2018-03-06

**Authors:** Gretchen L. Sacha, Simon W. Lam, Abhijit Duggal, Heather Torbic, Stephanie N. Bass, Sarah C. Welch, Robert S. Butler, Seth R. Bauer

**Affiliations:** 1 Department of Pharmacy, Cleveland Clinic, 9500 Euclid Avenue (Hb-105), Cleveland, OH 44195 USA; 2 Respiratory Institute, Cleveland Clinic, Cleveland, OH USA; 3 Department of Quantitative Health Sciences, Cleveland Clinic, Cleveland, OH USA

**Keywords:** Sepsis, Septic shock, Vasopressors, Vasopressin, Norepinephrine, Catecholamines

## Abstract

**Background:**

Vasopressin is often utilized for hemodynamic support in patients with septic shock. However, the most appropriate patient to initiate therapy in is unknown. This study was conducted to determine factors associated with hemodynamic response to fixed-dose vasopressin in patients with septic shock.

**Methods:**

Single-center, retrospective cohort of patients receiving fixed-dose vasopressin for septic shock for at least 6 h with concomitant catecholamines in the medical, surgical, or neurosciences intensive care unit (ICU) at a tertiary care center. Patients were classified as responders or non-responders to fixed-dose vasopressin. Response was defined as a decrease in catecholamine dose requirements and achievement of mean arterial pressure ≥ 65 mmHg at 6 h after initiation of vasopressin.

**Results:**

A total of 938 patients were included: 426 responders (45%), 512 non-responders (55%). Responders had lower rates of in-hospital (57 vs. 72%; *P* < 0.001) and ICU mortality (50 vs. 68%; *P* < 0.001), and increased ICU-free days at day 14 and hospital-free days at day 28 (2.3 ± 3.8 vs. 1.6 ± 3.3; *P* < 0.001 and 4.2 ± 7.2 vs. 2.8 ± 6.0; *P* < 0.001, respectively). On multivariable analysis, non-medical ICU location was associated with increased response odds (OR 1.70; *P* = 0.0049) and lactate at vasopressin initiation was associated with decreased response odds (OR 0.93; *P* = 0.0003). Factors not associated with response included APACHE III score, SOFA score, corticosteroid use, and catecholamine dose.

**Conclusion:**

In this evaluation, 45% responded to the addition of vasopressin with improved outcomes compared to non-responders. The only factors found to be associated with vasopressin response were ICU location and lactate concentration.

## Background

Due to its vasoconstrictive properties, arginine vasopressin (AVP) is often utilized in practice for patients with shock requiring hemodynamic support. The Surviving Sepsis Campaign guidelines suggest AVP as an adjunct to norepinephrine (NE) at a fixed dosage of 0.03 units/min to achieve mean arterial pressure (MAP) goals or decrease NE requirements [1]. However, due to limited data these recommendations have a weak grading. In the landmark Vasopressin and Septic Shock Trial (VASST), patients were randomized to either AVP plus NE or NE monotherapy, with no mortality difference detected between treatment approaches [2]. However, further analyses have suggested that patients with less severe forms of septic shock may benefit from AVP [2, 3]. Despite limited data supporting the efficacy of this agent and weak guideline recommendations, clinicians commonly utilize AVP in practice.

The importance of targeting and maintaining goal MAP along with early initiation of vasoactive agents in patients with septic shock has been associated with reduced mortality rates [4, 5]. In fact, delays in vasoactive initiation were associated with increased mortality [5]. Conversely, the importance of limiting catecholamines (CA) and utilizing non-CA vasoactive agents, such as AVP, is becoming more apparent and may ultimately improve patient outcomes [6–8]. Similarly, initiating AVP early in shock presentation may yield beneficial results [9, 10].

Unfortunately, there are still many unknowns regarding the most appropriate management strategy in patients with septic shock and the choice of vasoactive agent (especially second line) involves the weighing of a dynamic interplay of mechanisms and resultant responses of these agents. Specifically, one such agent is AVP and the ideal patient population to initiate AVP is unknown. There are limited data that may indicate a benefit in patients that are less severely ill [2, 3], have renal dysfunction [11, 12], or are receiving corticosteroids [13–15]. This study was designed to describe the impact of fixed-dose AVP on hemodynamic response and determine factors associated with response to AVP in a large cohort of adult patients with septic shock. The primary objective was to ascertain patient-specific factors at AVP initiation associated with a higher likelihood of response to AVP therapy. Secondary objectives included comparing clinical outcomes between responders and non-responders, and evaluating clinical characteristics over time, including MAP, lactate and CA dosage.

## Methods

This was a retrospective, single-center evaluation of fixed-dose AVP at a large tertiary care academic medical center. Adults over the age of 18 with active orders for AVP between September 2011 and August 2015 were screened for inclusion. Patients with septic shock, receiving adjunctive, fixed-dose AVP for at least 6 h in the medical intensive care unit (ICU), surgical ICU, or neurosciences ICU were included. Patients must have received one or more CA agent for at least 1 h prior to AVP initiation and only the first course of AVP was included. Patients were excluded if they had incomplete electronic data or AVP was initiated in the operating room.

Patients were classified as responders to AVP if they achieved both a decrease in CA dosage and MAP ≥ 65 mmHg 6 h after AVP initiation. Six hours was chosen based on an evaluation showing MAP during the first 6 h was independently associated with mortality in patients with septic shock [16]. CA dosage was described in NE-equivalent dosage requirements from the following formula [NE (mcg/min)] + [Epinephrine (mcg/min)] + [Dopamine (mcg/kg/min)/2] + [Phenylephrine (mcg/min)/10] [2]. Septic shock was defined as meeting two or more systemic inflammatory response syndrome criteria with the presence of antibiotics and hypotension requiring CAs. The presence of acute kidney injury (AKI) was determined and patients were categorized into one of the risk, injury, failure, loss, and end-stage kidney disease (RIFLE) categories based on serum creatinine increase at ICU admission and AVP initiation [11]. Total fluid bolus volume was calculated as crystalloid volume, with colloid equivalent doses [17, 18] and defined as total volume of fluids given 6 h prior to NE initiation until AVP initiation. Corticosteroid receipt was defined as receiving at least one dose of corticosteroids at AVP initiation up to 6 h after initiation.

Outcomes collected included in-hospital and ICU mortality, alive ICU-free days at day 14, alive hospital-free days at day 28, duration of mechanical ventilation, SOFA score change 48 h after AVP initiation, CA dosage change at 6 h after AVP initiation, need for continuous renal replacement therapy (CRRT) initiation, and CA duration. Cohorts of interest were defined a priori based on previous literature suggesting beneficial outcomes with AVP: NE-equivalent CA dose < 15 mcg/min at AVP initiation [2], lactate concentration ≤ 1.4 mmol/L at AVP initiation [2], receipt of corticosteroids [13, 15] obesity category [19, 20], the use of > 1 vasoactive agent at AVP initiation [2], and renal insufficiency per RIFLE category [11].

Data are presented as mean ± SD for continuous variables and *n* (%) for categorical variables. Univariate analyses between responders and non-responders were tested using either Chi-Square or Fisher’s exact test, as appropriate, for categorical variables or ANOVA for continuous variables. Between-group differences in change in MAP, lactate concentration, CA dosage requirements, and central venous oxygen saturation (ScvO_2_) were assessed at consecutive time intervals from AVP initiation to 72 h. A Bonferroni correction was applied to the pairwise comparisons. The effect of baseline variables on AVP response and ICU mortality were assessed using stepwise multivariable logistic regression. Statistically significant and variables with biologic plausibility for influencing the outcome were considered for the model and tested for colinearity using variance inflation factors and condition indices. If two variables were determined to be collinear [21], only one was included in the multivariable regression analysis. *P* values < 0.05 were considered to be statistically significant. All statistical analyses were performed with SAS 9.4 Software (SAS Institute Inc., Cary, NC) and StataIC 14 (StataCorp LLC, College Station, Tx). This study was approved by the Cleveland Clinic institutional review board (Study Number 15-2100).

## Results

**Fig. 1:**
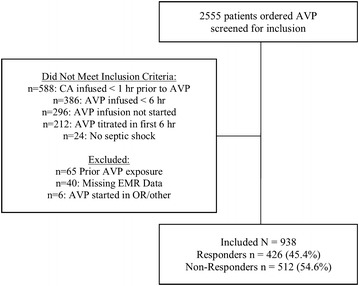
Patient inclusion and exclusion tree. There were 2555 patients screened for inclusion into the study. Of the screened patients, 1506 patients did not meet initial inclusion criteria and 111 met exclusion criteria leaving 938 patients included in the evaluation. *AVP* arginine vasopressin; *CA* catecholamine; *EMR* electronic medical record; *OR* operating room

Of the 2555 screened, 938 (36.7%) met criteria for inclusion and of these, 426 (45.4%) were classified as responders to AVP and 512 (54.6%) as non-responders (Fig. 1). The average age was 62 ± 14 years, most patients were Caucasian (69.5%) and treated in the medical ICU (75.9%; Table 1). When compared to responders, non-responders had higher rates of hepatic failure (19.3 vs. 14.3%; *P* = 0.04), lower MAP values (65 ± 12 vs. 69 ± 12 mmHg; *P* < 0.001) and higher lactate concentrations (5.4 ± 4.8 vs. 4.0 ± 3.6 mmol/L; *P* < 0.001) at AVP initiation. The average AVP initial dose was 0.03 units/min (range 0.01–0.08 units/min).

**Table 1:** Baseline characteristics

Characteristic	Total (*N* = 938)	Non-responders (*N* = 512)	Responders (*N* = 426)	*P* value
*Characteristics at ICU admission*				
Age, years	62 ± 14	61 ± 15	62 ± 14	0.17
Male, *n* (%)	493 (52.6)	272 (53.1)	221 (51.9)	0.70
Race, *n* (%)				0.10
Caucasian	652 (69.5)	357 (69.7)	295 (69.2)	
African American	241 (25.7)	124 (24.2)	117 (27.5)	
Other	45 (4.8)	31 (6.1)	14 (3.3)	
ICU type, *n* (%)				0.06
Medical	712 (75.9)	401 (78.3)	311 (73.0)	
Neurological	65 (6.9)	27 (5.3)	38 (8.9)	
Surgical	161 (17.2)	84 (16.4)	77 (18.1)	
Weight, kg	90.5 ± 34.0	92.0 ± 37.1	88.6 ± 29.9	0.13
BMI, kg/m^2^	31.5 ± 11.7	31.9 ± 12.6	31.0 ± 10.4	0.22
ESRD, *n* (%)	119 (12.7)	58 (11.3)	61 (14.3)	0.17
APACHE III	106 ± 34	107 ± 36	104 ± 30	0.09
APS	90 ± 32	92 ± 35	88 ± 29	0.14
Comorbid conditions, *n* (%)				
Diabetes mellitus	286 (30.5)	152 (29.7)	134 (31.5)	0.56
Hepatic failure	160 (17.1)	99 (19.3)	61 (14.3)	0.04
Immune suppression	196 (20.9)	109 (21.3)	87 (20.4)	0.75
Leukemia/myeloma	65 (6.9)	38 (7.4)	27 (6.3)	0.52
Moderate COPD	13 (1.4)	9 (1.8)	4 (0.9)	0.45
Severe COPD	85 (9.1)	42 (8.2)	43 (10.1)	0.45
No chronic health issues, *n* (%)	232 (24.7)	118 (23.0)	114 (26.8)	0.19
*Characteristics at time of AVP initiation*				
Appropriate antibiotics, *n* (%)^a^	887 (94.6)	487 (95.1)	400 (93.9)	0.41
Fluids prior to AVP, mL/kg	30.7 ± 34.4	30.6 ± 35.1	30.8 ± 33.6	0.95
MAP, mmHg	67 ± 12	65 ± 12	69 ± 12	< 0.001
Lactate, mmol/L	4.8 ± 4.4	5.4 ± 4.8	4.0 ± 3.6	< 0.001
SOFA score	13 ± 4	12 ± 3	13 ± 4	0.49
Total CA dose				
mcg/min	28.2 ± 19.9	27.8 ± 21.9	28.6 ± 17.3	0.54
mcg/kg/min	0.34 ± 0.26	0.33 ± 0.27	0.35 ± 0.25	0.18
Catecholamine agent, *n* (%)				
Norepinephrine	937 (99.9)	511 (99.8)	426 (100.0)	0.99
Phenylephrine	66 (7.0)	31 (6.1)	35 (8.2)	0.20
Epinephrine	25 (2.7)	11 (2.1)	14 (3.3)	0.28
Dopamine	4 (0.4)	2 (0.4)	2 (0.5)	0.99
AVP dose				
Units/min	0.0314 ± 0.0063	0.0317 ± 0.0064	0.0312 ± 0.0062	0.24
Units/kg/h	0.0226 ± 0.0084	0.0224 ± 0.0084	0.0227 ± 0.0083	0.66
Corticosteroids, *n* (%)	571 (60.9)	320 (62.5)	251 (58.9)	0.26
AKI, *n* (%)				0.21
Risk	79 (8.4)	50 (9.8)	29 (6.8)	
Injury	32 (3.4)	21 (4.1)	11 (2.6)	
Failure	142 (15.1)	75 (14.6)	67 (15.7)	
Loss	0 (0)	0 (0)	0 (0)	
CRRT, *n* (%)	159 (17.0)	81 (15.8)	78 (18.3)	0.31

*AKI* acute kidney injury, *AVP* arginine vasopressin, *APS* acute physiology score, *BMI* body mass index, *CA* catecholamine, *COPD* chronic obstructive pulmonary disease, *CRRT* continuous renal replacement therapy, *ESRD* end-stage renal dysfunction, *MAP* mean arterial pressure, *SOFA* sequential organ failure assessment

^a^Antibiotics were considered to be appropriate if patients received antibiotics described in the Centers for Medicare & Medicaid Services sepsis measure or received an appropriately de-escalated antibiotic regimen for an isolated pathogen on the day of AVP initiation

Responders had lower rates of in-hospital and ICU mortality (56.6 vs. 71.7%; *P* < 0.001 and 50.2 vs. 67.8%; *P* < 0.001, respectively), more ICU-free days at day 14 (2.3 ± 3.8 vs. 1.6 ± 3.3 days; *P* < 0.001), more hospital-free days at day 28 (4.2 ± 7.2 vs. 2.8 ± 6.0 days; *P* < 0.001) and less frequent need for CRRT within 72 h after AVP initiation (20.2 vs. 30%; *P* = 0.002) (Table 2). There was a significant difference between groups in the change in SOFA score from AVP initiation until 48 h (responders 0.30 ± 2.9 vs. non-responders 0.83 ± 2.9; *P* = 0.02) and CA dose change from AVP initiation until 6 h (responders − 12.8 ± 9.6 mcg/min vs. non-responders +13.8 ± 51.2 mcg/min; *P* < 0.001). Responders also had more CA-free and MV-free days on day 14 compared to non-responders (both *P* < 0.001). On multivariable logistic regression, treatment in the surgical or neurosciences ICU compared to the medical ICU and lower lactate concentrations was independently associated with higher odds of response to AVP (*P* = 0.005 and *P* < 0.001, respectively). Additionally, a positive hemodynamic response to AVP was independently associated with lower ICU mortality (Table 3).

**Table 2:** Patient outcomes

Outcome	Total (*N* = 938)	Non-responders (*N* = 512)	Responders (*N* = 426)	*P* value
In-hospital mortality, *n* (%)	608 (64.8)	367 (71.7)	241 (56.6)	< 0.001
ICU mortality, *n* (%)	561 (59.8)	347 (67.8)	214 (50.2)	< 0.001
ICU-free days at day 14	1.9 ± 3.6	1.6 ± 3.3	2.3 ± 3.8	< 0.001
Hospital-free days at day 28	3.4 ± 6.6	2.8 ± 6.0	4.2 ± 7.2	< 0.001
MV-free days at day 14	2.8 ± 4.9	2.2 ± 4.5	3.6 ± 5.3	< 0.001
SOFA score change^a^	0.6 ± 2.9	0.8 ± 2.9	0.3 ± 2.9	0.02
Respiration score change	2.3 ± 1.5	2.0 ± 1.5	2.5 ± 1.4	< 0.001
Coagulation score change	0.46 ± 1.0	0.5 ± 0.9	0.4 ± 1.0	0.19
Liver score change	0.1 ± 0.7	0.1 ± 0.8	0.7 ± 0.6	0.90
Neurological score change	− 0.1 ± 1.1	0.1 ± 1.1	− 0.2 ± 1.0	< 0.001
Cardiovascular score change	− 1.9 ± 1.7	− 1.6 ± 1.7	− 2.1 ± 1.7	< 0.001
CRRT initiation between AVP start and 72 h, *n* (%)^b^	190 (25.0)	112 (30.0)	78 (20.2)	0.002
CA dose change^c^, mcg/min	+1.7 ± 40.6	+13.8 ± 51.2	− 12.8 ± 9.6	< 0.001
CA-free days at day 14	5.0 ± 5.8	3.9 ± 5.5	6.3 ± 6.0	< 0.001

*CA* catecholamine, *CRRT* continuous renal replacement therapy, *MV* mechanical ventilation, *SOFA* sequential organ failure assessment

^a^Evaluated at hour 48 after vasopressin initiation

^b^Evaluated only in patients who survived at least 24 h after vasopressin initiation

^c^Evaluated at hour 6 after vasopressin initiation

**Table 3:** Results of multivariable analyses

Outcome	OR (95% CI)	*P* value
Multivariable analysis and association with response to vasopressin^a^		
Non-medical ICU	1.70 (1.18–2.46)	0.005
Lactate at AVP initiation, mmol/L	0.93 (0.89–0.97)	< 0.001
Multivariable analysis and association with ICU mortality		
Hemodynamic response to AVP	0.51 (0.35–0.76)	0.001
Catecholamine dose, mcg/kg/min	3.14 (1.36–7.28)	0.008
Lactate at AVP initiation, mmol/L	1.10 (1.04–1.18)	0.002
AKI presence		
Rifle versus no AKI	3.64 (1.77–7.49)	< 0.001
Injury versus no AKI	5.80 (1.13–29.60)	0.035
Failure versus no AKI	2.63 (1.38–5.01)	0.003
ESRD versus no AKI	2.37 (1.27–4.43)	0.007
APACHE III score	1.01 (1.01–1.02)	< 0.001
SOFA score	1.16 (1.08–1.25)	< 0.001
Medical ICU	1.58 (1.02–2.45)	0.040
Race (Caucasian)	1.72 (1.14–2.60)	0.010
Age	1.01 (1.00–1.03)	0.036
Hepatic failure	0.89 (0.48–1.62)	0.696

*AKI* acute kidney injury, *AVP* vasopressin, *ESRD* end-stage renal dysfunction, *SOFA* sequential organ failure assessment

^a^Variables entered into the model but without a statistically significant association with vasopressin response include RIFLE-defined AKI category, corticosteroid use, SOFA score, APACHE III score, hepatic failure, race, age, and catecholamine dosage (in mcg/kg/min)

In the predefined cohorts of interest, there was no association between the cohort designation and hemodynamic response in patients whether classified on the receipt of corticosteroids, obesity category, number of vasopressors required at AVP initiation, or RIFLE-defined AKI. Patients with lactate concentrations ≤ 1.4 mmol/L had higher odds of response to AVP while patients with NE-equivalent CA doses < 15 had a decreased odds of response to AVP (Table 4).

**Table 4:** Predefined cohorts of interest

Cohort of interest	Responders *N* (%)	Non-responders *N* (%)	*P* value	OR (95% CI) hemodynamic response	OR (95% CI) ICU mortality
*Corticosteroids*					
Yes	251 (58.9)	320 (62.5)	0.26	0.86 (0.66–1.12)	1.01 (0.77–1.32)
No^a^	175 (41.1)	192 (37.5)			
*Lactate concentration*					
> 1.4 mmol/L^a^	211 (78.4)	321 (88.7)	< 0.001	2.15 (1.39–3.32)^^^	0.39 (0.25–0.60)^^^
≤ 1.4 mmol/L	58 (21.6)	41 (11.3)			
*BMI classification*					
Underweight^a^	18 (4.2)	20 (3.9)	0.98		
Normal	94 (22.1)	106 (20.7)		0.99 (0.50–1.97)	1.09 (0.53–2.12)
Overweight	114 (26.8)	140 (27.3)		0.90 (0.46–1.79)	1.02 (0.51–2.05)
Obesity class I	81 (19.0)	101 (19.7)		0.89 (0.44–1.80)	0.91 (0.45–1.86)
Obesity class II	48 (11.3)	53 (10.4)		1.01 (0.48–2.12)	1.13 (0.52–2.43)
Obesity class III	71 (16.7)	92 (18.0)		0.86 (0.42–1.74)	0.77 (0.37–1.57)
*CA equivalent dose*					
≥ 15 mcg/min^a^	370 (86.9)	424 (82.8)	0.087	0.57 (0.36–0.92)^^^	0.62 (0.44–0.89)^^^
< 15 mcg/min	56 (13.1)	88 (17.2)			
*Total vasopressor quantity*					
1 Vasopressor^a^	370 (86.9)	463 (90.4)	0.084	1.43 (0.95–2.15)	1.98 (1.26–3.12)^^^
> 1 Vasopressor	56 (13.1)	49 (9.6)			
*Renal insufficiency*					
Yes	220 (51.6)	268 (52.3)	0.83	0.97 (0.75–1.26)	1.59 (1.23–2.07)^^^
No^a^	206 (48.4)	244 (47.7)			
*AKI class*					
No AKI presence^a^	258 (60.6)	308 (60.2)	0.21		
AKI-risk	29 (6.8)	50 (9.8)		0.69 (0.43–1.13)	2.40 (1.43–4.03)^^^
AKI-injury	11 (2.6)	21 (4.1)		0.63 (0.30–1.32)	3.30 (1.41–7.76)^^^
AKI-failure	67 (15.7)	75 (14.6)		1.07 (0.74–1.54)	3.06 (2.00–4.66)^^^
AKI-end stage	61 (14.3)	58 (11.3)		1.26 (0.85–1.87)	1.64 (1.09–2.46)^^^

*AKI* acute kidney injury, *BMI* body mass index, *CA* catecholamine

^^^*P* < 0.05

^a^The reference group used for the odds ratio result

**Fig. 2:**
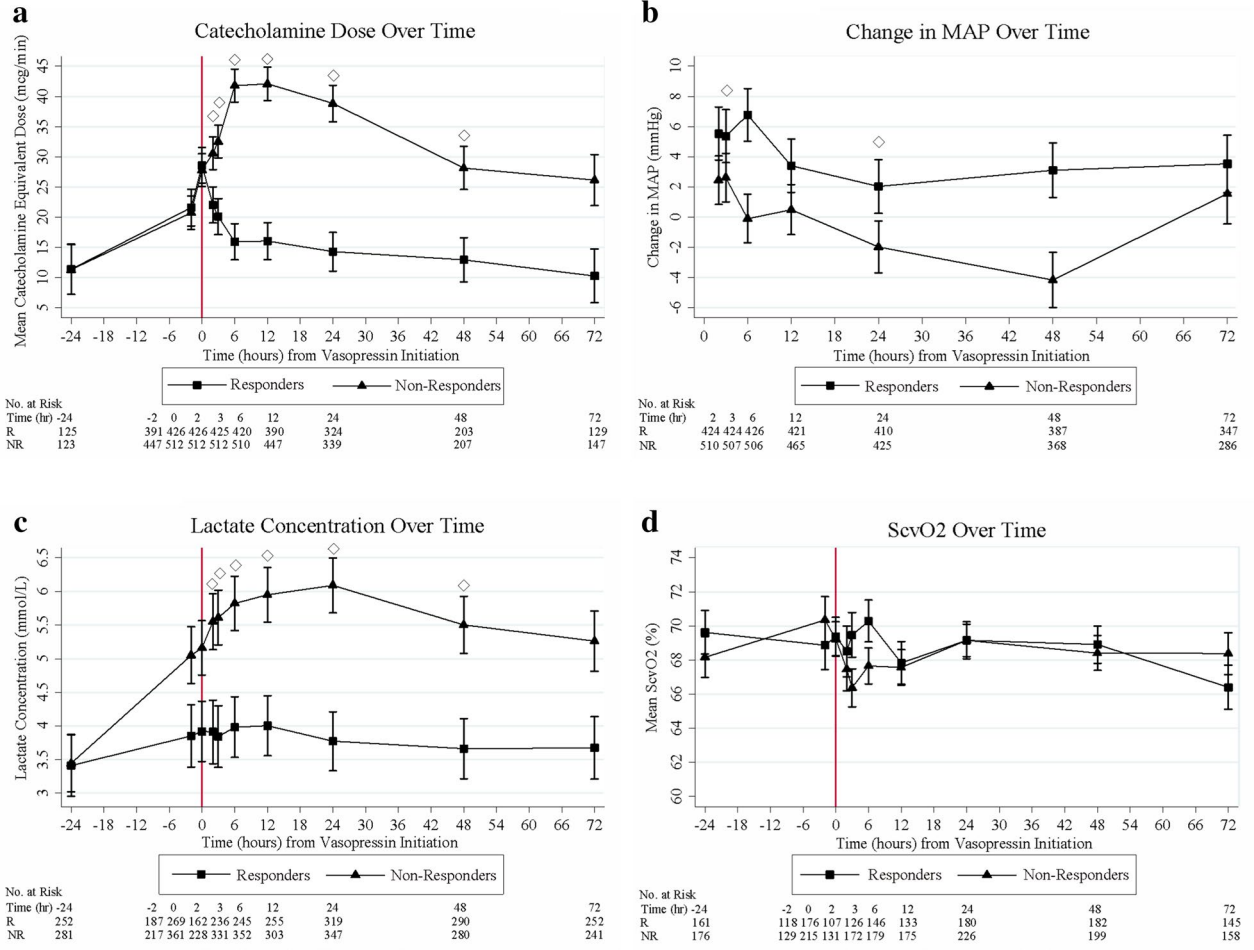
Patient results over time for vasopressin responders and non-responders. **a** Catecholamine dose from -24 h to 72 h after vasopressin initiation. Responders had significantly lower catecholamine doses at 2, 3, 6, 12, 24 and 48 h after vasopressin initiation compared to non-responders. **b** Change in MAP from time 0 to 72 h after vasopressin initiation. Responders had significantly higher degrees of MAP change at 3 and 24 h after vasopressin initiation compared to non-responders. **c** Changes in lactate concentration from -24 h to 72 h after vasopressin initiation. Responders had significantly lower lactate concentrations at 2, 3, 6, 12, 24, and 48 h compared to nonresponders. **d** ScvO_2_ from -24 h to 72 h after vasopressin initiation. There was no difference in ScvO_2_ between responders and non-responders at any time point evaluated. *MAP* mean arterial pressure; *NR* non-responders; *R* responders; *ScvO2* central venous oxygen saturation. Data are means, with error bars indicating standard deviation. ◊ P < 0.001

There was a significant difference in CA dosage between responders and non-responders at every time point from AVP initiation through 48 h (Fig. 2a). There was also a significant difference in MAP change from AVP initiation in the responders compared to the non-responders at 3 and 24 h: +5.4 versus +2.6 mmHg (*P* < 0.001) and +2.0 versus − 2.0 mmHg (*P* < 0.001) (Fig. 2b). Finally, lactate concentration differed significantly between responders and non-responders at every time point evaluated from AVP initiation through 48 h (Fig. 2c). There was no difference in ScvO_2_ at any time point (Fig. 2d).

## Discussion

This evaluation identified 938 patients in which 45% had a positive hemodynamic response to AVP which was associated with decreased mortality, increased ICU- and hospital-free days, and decreased CA dosage requirements. The improvement in outcomes in responders indicates the definition used for hemodynamic response may be an appropriate pharmacodynamic marker of response to AVP therapy and should be further evaluated in future studies. Furthermore, on multivariable analyses, non-medical ICU treatment and decreasing lactate concentrations were independently associated with a positive response to AVP and AVP response was independently associated with decreased ICU mortality. It is important to understand that the clinical utilization of AVP and its place in therapy relies on imperfect data, clinical experience, and weak guideline recommendations. Regardless of this, it is commonly used in clinical practice as an adjunct to NE in patients with refractory septic shock [22]. Its proposed mechanism of action is twofold, by causing V1 receptor-mediated vasoconstriction in some vascular smooth muscle beds [23], AVP can be utilized as a vasopressor similar to CAs. Additionally, in patients with septic shock, a relative endogenous vasopressin deficiency may exist and fixed, low dose exogenous AVP can be utilized as an endocrine supplement with resultant improvements in hemodynamics [12, 24–27]. Clinicians are often put in challenging situations in which they must determine if AVP should be initiated for an individual patient with few data available to inform the decision. The results of this study identify patient characteristics associated with response to AVP and can assist with decision-making regarding AVP initiation.

VASST is the largest trial of AVP in septic shock and randomized patients to either AVP plus NE or NE monotherapy [2]. While no mortality difference was detected between groups in the main analysis, several subsequent analyses have suggested benefit in specific subgroups of patients. In a priori-defined subgroup analyses, VASST showed improved 28- and 90-day outcomes in patients allocated to AVP with “less severe septic shock” (CA requirements < 15 mcg/min) and patients receiving one vasopressor at baseline (compared to two or more) [2], findings which were not corroborated in the current study. Furthermore, in contrast to VASST, the current study found patients with CA doses < 15 mcg/min had lower odds of response to AVP. The cutoff of 15 mcg/min was based on the results of VASST; however, it is unknown if an optimal CA dose threshold for achieving hemodynamic response with AVP exists and 15 mcg/min may not be the ideal threshold to evaluate. In fact, in a recent retrospective cohort study, increasing the AVP initiation threshold from a NE dose of 10 mcg/min to 50 mcg/min was not associated with increased mortality [28]. It should be noted that in clinical practice, AVP is frequently initiated in patients with NE dosage requirements exceed 15 mcg/min. In fact, the average NE dose at AVP initiation was 28 mcg/min in the current study which is similar to VASST (20 mcg/min) [2].

In an additional VASST post hoc subgroup analysis, patients receiving AVP with baseline lactate concentration ≤ 1.4 mmol/L had lower 28-day mortality rates than those receiving NE [2]. A subsequent re-analysis of VASST based on the updated definitions for septic shock also found improved survival in patients initiated on AVP with a lactate concentration ≤ 2 mmol/L [3]. The current study parallels these findings, with lower lactate concentrations independently associated with higher odds of hemodynamic response. Altogether, low lactate concentrations appear to be a useful biomarker for initiation of AVP. In comparison with VASST, which found no effect on renal replacement therapy, the current evaluation showed that fewer responders required a new initiation of CRRT compared to non-responders. These findings corroborate those from the Vasopressin versus Norepinephrine as Initial Therapy in Septic Shock (VANISH) trial which showed a decreased rate of renal replacement therapy initiation in patients who received vasopressin (when compared to NE) [29].

In addition, this study found no association with corticosteroid use and hemodynamic response; a combination previously thought to have a positive interaction [13]. The lack of an effect observed in this evaluation compared to previous studies could be due to differences of corticosteroid use. In the VASST analysis, the use of corticosteroids was regarded as receipt of at least one dose within the 28-day observation period, whereas the current study ensured corticosteroids were used concomitantly with AVP. However, it is important to note that patients could have received corticosteroids up to 6 h after AVP initiation, potentially affecting their ability to detect a response to corticosteroids in the evaluated time frame. Furthermore, the lack of detected benefit with corticosteroids could be due to differences in the outcomes evaluated in the current study (hemodynamic response) versus historical studies (mortality) [2, 13–15]. However, the lack of association seen in the current evaluation corroborates the findings seen in VANISH which detected no interaction between AVP and corticosteroid use on 28-day mortality [29]. Additional studies are needed to determine the relationship between corticosteroid use and hemodynamic response to AVP in patients with septic shock.

An additional finding of the current study was the CA-sparing effect, in that CA dosages decreased in responders at every time point from AVP initiation until 48 h. In fact, because the MAP was > 65 mmHg when AVP was added, a CA-sparing effect was likely the intended goal of AVP initiation. Responders also had more CA-free days at day 14 compared to non-responders, further showing the CA-sparing effect observed in this group. The benefit of sparing CAs in patients with septic shock has recently become more apparent [6–8]. One analysis found that raising MAP values above 70 mmHg with increasing vasoactive doses resulted in increased organ failure events [6]. Additionally, excess CAs can have a negative effect on the immune system and can cause tachyarrhythmias, hyperglycemia, splanchnic hypoperfusion, and myocardial depression. This new perspective emphasizes the importance of limiting CA doses while maintaining goal MAP, a method that can be achieved through AVP utilization.

Upon multivariable logistic regression, treatment in the medical ICU was associated with lower odds of response to AVP. Patients with sepsis secondary to medical (vs. surgical) conditions have higher mortality [30], which may influence AVP response. Additionally, these patient populations can present with a differing mix of comorbidities, which may alter patient outcomes differently [31], and medical patients may have lower frequencies of infectious source control (due to the prevalence of in-operable infections, i.e., pneumonia), which could decrease their response to treatment, including vasoactive therapies. It is also possible that there were residual confounders between medical ICU and non-medical ICU patients unable to be controlled for in the multivariable model. This finding of differing AVP response by treatment ICU and the potential mechanisms should be explored further.

This study has important implications for practice and future research. Regardless of the patients’ CA dose, the association between low lactate concentration and hemodynamic response with AVP suggests that this marker of “less severe septic shock” is a useful indicator for AVP initiation. Furthermore, because of the improved outcomes in patients who had a positive hemodynamic response to AVP at 6 h, monitoring for the achievement of hemodynamic stability can be an important early warning sign for the bedside clinician. Specifically, in patients who do not achieve hemodynamic stability within 6 h of starting AVP, alternative therapeutic interventions such as epinephrine [32], corticosteroids [33, 34], angiotensin II [35] (if available), or increasing AVP dose (especially when NE requirements exceed 0.6 mcg/kg/min) [36] should be considered. The use of this trigger and the next best step should be further investigated. Future trials should incorporate the observed factors associated with AVP response into their design, which may improve their likelihood of finding a target population for AVP use. Additionally, trials should evaluate when to initiate additional adjunctive agents and also compare efficacy between adjunctive agents.

Strengths of this evaluation include its a priori-defined cohorts for analysis, and evaluation of fixed-dose AVP (which removes the potential confounder of titrated doses on AVP response). Limitations of this evaluation include the fact that it was a single-center, retrospective study with no randomization and relied on medical record charting that may not instantaneously capture exact medication administration timing and hemodynamic change. Secondly, the definition of AVP response was not developed based on previous literature or able to be validated in this current study, but was created in an attempt to reflect hemodynamic response to this agent. However, based on the observed differences between responders and non-responders, it appears to accurately reflect a clinically meaningful response. Albeit, with this definition, patients who were already in the recovery phase of septic shock with decreasing CA dosage at AVP initiation were regarded as “responders.” Additionally, patients classified as “non-responders” may have had decreased overall CA exposure with AVP than if they were not started on AVP, which was not accounted for in our definition of response. This study was also unable to incorporate markers of tissue perfusion (e.g., lactate, urine output, pH) into the definition of hemodynamic response, because these parameters were not consistently or frequently monitored and documented for every included patient. The importance of markers of tissue perfusion should not be overlooked as patients could potentially be at goal MAP, with reductions in CA doses as a result, but still have tissue hypoperfusion. This study also classified patients as having septic shock based on the previous definition and not the updated 2016 definition [1] which may result in more patients being included than those who had septic shock per the newest definition. Furthermore, cardiac output data were not available for most patients and therefore not collected. Although ScvO_2_ values were elevated at baseline and not significantly different between responders and non-responders, we cannot adequately compare cardiac output between response groups. Additionally, the retrospective nature of this study makes identifying patients with true septic shock difficult, and as such, patients may have been included or excluded inadvertently. Finally, excluding patients who did not receive AVP for at least 6 h may have influenced the rates of response to therapy, as there may have been patients who responded earlier than 6 h and no longer needed vasoactive support with AVP (true responders) or patients who died within 6 h (true non-responders) and subsequently were excluded from the evaluation.

## Conclusion

The current evaluation identified a large cohort of patients receiving fixed-dose AVP in which 45% responded to therapy. AVP response was associated with improved mortality and ICU and hospital-free days, indicating the definition used for hemodynamic response may be an appropriate pharmacodynamic marker of AVP therapy that can be used in future trials. In agreement with historical trials, patients with less severe forms of septic shock (lower lactate concentrations at baseline) appear to benefit more from AVP in comparison with patients with more severe forms. Future studies should incorporate the observed factors related to AVP response into their subsequent design to definitively identify the most appropriate patient population that would benefit from AVP.

## Abbreviations

AKI: acute kidney injury
AVP: arginine vasopressin
CA: catecholamine
CRRT: continuous renal replacement therapy
ICU: intensive care unit
NE: norepinephrine
MAP: mean arterial pressure
RIFLE: risk, injury, failure, loss, end-stage kidney disease category
ScVO_2_: central venous oxygen saturation
SOFA: sequential organ failure assessment
VASST: vasopressin and septic shock trial
